# Authentication of *Nigella sativa* Seed Oil in Binary and Ternary Mixtures with Corn Oil and Soybean Oil Using FTIR Spectroscopy Coupled with Partial Least Square

**DOI:** 10.1155/2013/740142

**Published:** 2013-11-11

**Authors:** Abdul Rohman, Rizka Ariani

**Affiliations:** ^1^Department of Pharmaceutical Chemistry, Faculty of Pharmacy, Gadjah Mada University, Yogyakarta 55281, Indonesia; ^2^Center of Research for Fiqh Science and Technology (Cfirst), Universiti Teknologi Malaysia, Skudai 80200, Malaysia; ^3^Research Center of Halal Products, Gadjah Mada University, Yogyakarta 55281, Indonesia

## Abstract

Fourier transform infrared spectroscopy (FTIR) combined with multivariate calibration of partial least square (PLS) was developed and optimized for the analysis of *Nigella* seed oil (NSO) in binary and ternary mixtures with corn oil (CO) and soybean oil (SO). Based on PLS modeling performed, quantitative analysis of NSO in binary mixtures with CO carried out using the second derivative FTIR spectra at combined frequencies of 2977–3028, 1666–1739, and 740–1446 cm^−1^ revealed the highest value of coefficient of determination (*R* ^2^, 0.9984) and the lowest value of root mean square error of calibration (RMSEC, 1.34% v/v). NSO in binary mixtures with SO is successfully determined at the combined frequencies of 2985–3024 and 752–1755 cm^−1^ using the first derivative FTIR spectra with *R* ^2^ and RMSEC values of 0.9970 and 0.47% v/v, respectively. Meanwhile, the second derivative FTIR spectra at the combined frequencies of 2977–3028 cm^−1^, 1666–1739 cm^−1^, and 740–1446 cm^−1^ were selected for quantitative analysis of NSO in ternary mixture with CO and SO with *R* ^2^ and RMSEC values of 0.9993 and 0.86% v/v, respectively. The results showed that FTIR spectrophotometry is an accurate technique for the quantitative analysis of NSO in binary and ternary mixtures with CO and SO.

## 1. Introduction

*Nigella sativa* L. also known as black cumin has been used for centuries, especially in the Middle East and Southeast Asia. The effects of *N. sativa* seed have been studied by many researchers. *N. sativa* seed has a broad spectrum as antibacterial [1–3], antitumor [4], anti-inflamatory [5], depressant of central nervous system, analgesic [6], and hypoglycemic [7] and smooth muscles relaxant [8–10], as well as cytotoxic and immunostimulant [11]. *Nigella sativa* oil (NSO) has been widely distributed in Arab countries and other parts of the Mediterranean region. NSO contains a large number of active compounds and has beneficial health effects like protective and curative activities. In the market, NSO has been sold 10–15 times more expensive than other edible oils such as corn oil (CO) and soybean oil (SO). This leads to the frequent occurrence of NSO to be blended with cheaper oils in order to get the maximum profits [12]. 

Adulteration of foodstuffs including oil is a serious problem because of the dangerous effects that may arise from additional ingredients that mixed into foods, such as the emergence of an allergic reaction [13]. Besides, the mixing of incompatible materials can also be associated with restrictions by certain religion, like adulteration oils with lard. Several methods such as high performance liquid chromatography (HPLC) [14], carbon isotope ratio [15], and electronic nose [16] have been developed to detect the adulteration of edible oils. However, those methods are time-consuming, expensive, generally destructive to the sample material, and too laborious. Therefore, a rapid and simple technique such as FTIR spectroscopy is used for routine monitoring of oil adulteration.

FTIR spectroscopy combined with multivariate calibration is a rapid and reliable technique for quantitative analysis of oils in mixture. Multivariate calibration is an analysis that uses several variables (absorbances in many wavenumbers) and is often used for the analysis of complex mixture [17]. One of the most commonly used multivariate calibration methods is partial least square (PLS). With PLS, it is possible to extract subtle information from complex spectra that contain overlapping and interference peaks and instrumental artifacts due to measurement condition [18].

In authentication study, FTIR spectroscopy in combination with multivariate calibration has been used for authentication of extra virgin olive oil from palm oil [19], authentication of olive oil from some vegetable oils [20, 21], and classification and quantification of corn oil and sunflower oil in olive oil [22], as well as for authentication of *Nigella sativa* oil from grape seed oil [12]. Using the literature review, there is no report regarding the use of FTIR spectroscopy for the analysis of corn oil (CO) and soybean oil (SO), having similar FTIR profile based on principal component analysis. In this study, FTIR spectroscopy combined with multivariate calibration of PLS was optimized for the determination of NSO in binary and ternary mixture systems with CO and SO.

## 2. Materials and Methods

### 2.1. Materials

*Nigella* seed oil (NSO), corn oil (CO), and soybean oil (SO) were purchased from supermarket in Yogyakarta, Indonesia. The standard of fatty acid methyl esters (FAMEs) of 37 compounds was bought from Sigma Chemicals (St. Louis, MO, USA). All chemicals and reagents used were of analytical grade.

### 2.2. Fatty Acid Analysis

In order to assure the authenticity of the used oils (NSO, CO, and SO), their fatty acid (FA) compositions were determined using a gas chromatograph (Shimadzu GC-2010, Shimadzu Corp., Tokyo, Japan), equipped with flame ionization. Before being analyzed, the samples of oils were derivatized using sodium methoxide to form FAMEs according to the method described by the American Oil Chemists Society (AOCS) [23]. The column used during gas chromatography analysis is RTX-5 (30 m × 0.25 mm, layer thickness 0.2 *μ*m), Restek Corp., Bellefonte, PA, USA. The initial temperature was 50°C (hold for 1 min), then ramped into 200°C (8°C/min), and finally held at 200°C for 5 min. The carrier gas of N_2_ was delivered at flow rate of 6.8 mL/min. The temperatures of detector and injector were maintained at 200°C, with spliting ratio of 1 : 20. The qualitative analysis of FAMEs in the samples was carried out by comparing retention times of the peaks with those of FAMEs standards. Quantification of individual fatty acids was performed using the technique of internal normalization and expressed as percentage based on peak area. This technique is done by dividing each component of FAME peaks with a total area of all peaks in the FAME chromatogram.

### 2.3. Preparation of Oil Samples

The calibration samples composed of NSO in binary and ternary mixtures with CO and SO in the concentration range 0–100% (v/v) was prepared. Furthermore, a series of independent samples was also prepared as validation samples in order to evaluate the predictive ability of the developed calibration model. 

### 2.4. FTIR Spectra Measurements

FTIR spectra of all evaluated samples were acquired using FTIR spectrophotometer ABB MB3000 (Canada) equipped with ZnSe crystal, with sample handling technique of attenuated total reflectance (ATR), using detector of deuterated triglycine sulfate (DTGS), and connected to Horizon MB software. Samples are placed on the ATR crystal with restrained temperature (20°C). The collection of FTIR spectra was carried out at 32 scans with resolution of 8 cm^−1^ in the frequency regions of 4000–650 cm^−1^. After every scan, a new reference air background spectrum was taken. The ATR plate was carefully cleaned using hexane twice followed by acetone and dried with a soft tissue before filling with the next samples. These spectra were recorded as absorbance values, and replication was done 2 times.

### 2.5. Multivariate Data Analysis

The software Horizon MB (Canada) was used during performing multivariate calibration. Spectral regions that showed FTIR spectra difference between NSO, CO, and SO were selected to make PLS model. Worksheet Excel 2010 was used to correlate between actual concentration and predicted concentration. The performance of the obtained calibration model was evaluated using the values of coefficient of determination (*R* ^2^) and root mean square error of calibration (RMSEC). As for the calibration, the performance of the obtained validation model was evaluated using the values of *R* ^2^ and root mean square error of prediction (RMSEP). 

## 3. Result and Discussion

### 3.1. Spectral Analysis

**Figure 1:**
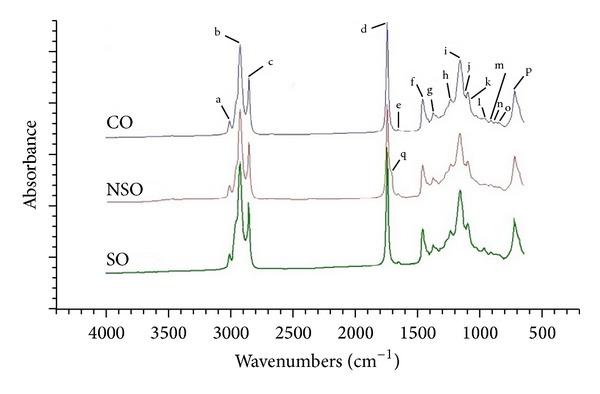
FTIR spectra of *Nigella sativa* L. seed oil, corn oil, and soybean oil at mid-infrared region range of 4000–650 cm^−1^.

Figure 1 showed FTIR spectra of authentic NSO, CO, and SO at mid-IR regions of 4000–650 cm^−1^. Each band and shoulder in FTIR spectra corresponds to functional groups responsible for infrared absorption and exhibits the characteristic bands for edible fats and oils [24]. The entire range of spectra for NSO, CO, and SO look very similar to the naked eyes. However, due to the fingerprint technique, meaning that there is no two compounds or samples having the same spectra in terms of amount and intensity of peaks, FTIR spectroscopy can be used to extract the differences among these oils. 

**Figure 2:**
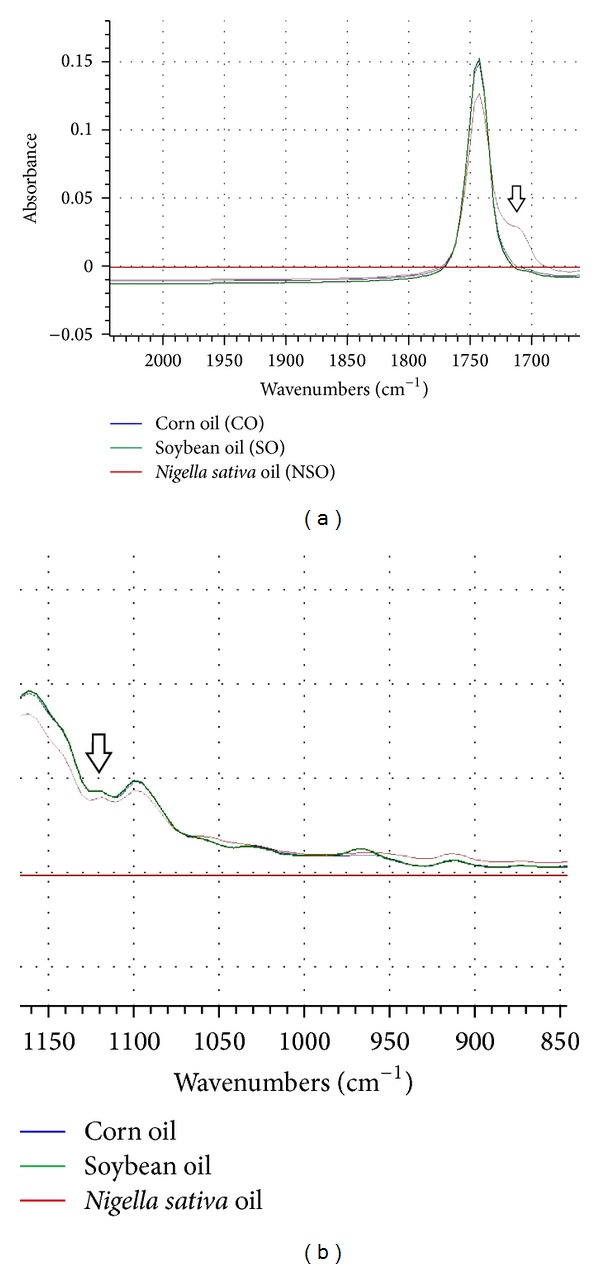
The magnified FTIR spectra frequency region of 1750–1700 cm^−1^ (a) and at 1128–1084 cm^−1^ (b). *x*-axis = wavenumbers (cm^−1^), and *y*-axis = absorbance.

If one examines the spectra closely, they reveal some differences which can be observed in the region around 1750–1700 cm^−1^ (peak d). NSO has two peaks at frequency region of 1750–1700 cm^−1^, meanwhile CO and SO revealed one peak. These peaks were attributed to carbonyl C=O stretching vibration from the ester linkage of triacylglycerol. Furthermore, at frequency region of 1128–1084 cm^−1^ (peak j and k), NSO also has two peaks; meanwhile, CO and SO appear with one peak. The magnified FTIR spectra at frequency region of 1750–1700 cm^−1^ and at 1128–1084 cm^−1^ were shown in Figure 2. These peaks were attributed to C–O stretching vibration. These peak intensity differences can be exploited for the quantification of NSO, CO, and SO in complex mixtures. The functional group responsible for IR absorption in the evaluated oils is compiled in Table 1.

FTIR spectra of NSO, CO, and SO are very similar since the main components of oils are triglycerides with certain fatty acids (Table 2). Analysis of fatty acid composition revealed that the used oils (NSO, Co, and SO) have similar profile of fatty acids as stated in Codex [25]. In the case of mixtures, one of the major difficulties is the interference and overlapping of the absorption bands that appear in FTIR spectra. The spectral differences are not easily detectable by univariate analysis. For this reason, a multivariate calibration of PLS regression using several absorbances in selected frequency regions as variables was used to overcome this problem. 

During developing PLS regression, the samples were divided into the calibration and the validation sets. In the PLS model, evaluation of the method linearity was performed to show the proportional relationship between responses (absorbance) versus analyte concentrations of NSO in the mixtures over the working range [26]. The spectral regions exploited for PLS analysis are optimized in such a way that gives higher value of *R* ^2^ and lowest value of RMSEC and RMSEP. The higher value of *R* ^2^ and the lower value of RMSEC and RMSEP indicated the better PLS model. Besides, the FTIR spectra were also subjected to derivatization using Savitzky-Golay first and second derivatives [27]. The first derivative can remove the common intensity effect of FTIR spectra and can simplify the baseline selection. Meanwhile, the second derivative can eliminate the slope effect. However, derivation treatments can strongly affect the analytical sensitivity [28, 29].

### 3.2. Quantitative Analysis of NSO in Binary Mixture with CO

Based on the highest value of *R* ^2^ and the lowest value of RMSEC as shown in Table 3, the combined frequencies region of 2977–3028, 1666–173, and 740–1446 cm^−1^ was selected for quantification of NSO in binary mixture with CO. PLS that shows the highest value of *R* ^2^ (0.9984) and the lowest value of RMSEC (1.33%) was selected and used for quantification of NSO in validation model. The second derivative FTIR spectra were exploited for determination of NSO. This result can be extended such as the adulterated NSO can be monitored using second derivative spectra at frequency regions of 2977–3028, 1666–1739, and 740–1446 cm^−1^ with the highest value of *R* ^2^ (0.9987) and the lowest value of RMSEP (0.66% v/v). Table 3 compiled the performance of PLS in term of *R* ^2^, RMSEC, and RMSEP as well as the equation for the relationship between actual and predicted values for determination of NSO in binary mixture with CO, either in calibration or validation data sets.

### 3.3. Quantitative Analysis of NSO in Binary Mixtures with SO

The calibration performance of PLS for quantification of NSO in binary mixture with SO is shown in Table 4. PLS using first derivative spectra at combined frequancies of 2985–3024 and 752–1755 cm^−1^ shows the highest value of *R* ^2^ (0.997) and the lowest value of RMSEC (0.47% v/v) for the prediction of NSO in binary mixture with SO. The equation obtained for the relationship between actual and FTIR predicted values in calibration data set was *y* = 0.9998*x* + 0.0702.

The PLS calibration model was further used to analyze the validation samples. From the results obtained, it can be shown that PLS using first derivative spectra at frequency region of 2985–3024 and 752–1755 cm^−1^ is the better model, in terms of the highest value of *R* ^2^ (0.9988) and the lowest value of RMSEP (0.63%) for the relationship between actual value (*x*-axis) and FTIR predicted (*y*-axis) of NSO in binary mixture with SO. The PLS regression model appears to have a reasonable ability to estimate the NSO percentage in the mixture with SO, based on the high *R* ^2^ and low RMSEC. 

### 3.4. Quantitative Analysis of NSO in Ternary Mixtures with CO and SO

PLS model has been done at certain frequencies, including those selected for analysis of  NSO in binary mixture with CO or SO. The calibration performance of PLS for quantification of NSO in ternary mixture with CO and SO is shown in Table 5. PLS using second derivative spectra at combined frequencies of 2977–3028, 1666–1739, and 740–1446 cm^−1^ shows the highest value of *R* ^2^ (0.9993) and the lowest value of RMSEC (0.86% v/v). The equation obtained for the relationship between actual and FTIR predicted values of NSO in ternary mixture with CO and SO in calibration data set was *y* = 1.0039*x* − 0.3253. 

The PLS calibration model was further used to analyze the validation samples. From the results obtained, PLS using second derivative spectra at frequency region of 2977–3028, 1666–1739, and 740–1446 cm^−1^ can predict well the validation sample due to the highest value of *R* ^2^ (0.9985) and the lowest value of RMSEP (0.58% v/v). Based on the highest value of *R* ^2^ and the lowest value of RMSEC, FTIR spectroscopy combined with multivariate PLS regression in certain frequencies is an accurate technique for the quantification of NSO in binary or ternary mixture with CO and SO. In addition, the low values of RMSEC and RMSEP indicated that the developed method is reproducible enough.

## 4. Conclusion

In conclusion, FTIR spectroscopy combined with chemometrics of PLS regression is a powerful technique for the quantitative analysis of NSO in binary and ternary mixtures with CO and SO. The developed method is fast, does not need excessive sample preparation, and does not involve the use of reagents and chemicals.

## Figures and Tables

**Table 1:** Functional groups and mode of vibration from FTIR spectra of the evaluated oils [19, 20].

Marker	Peak position of FTIR spectra (cm^−1^)	Assignment of bands	Mode of vibration
(a)	3009	C=CH (*cis*)	Stretching
(b) and (c)	2922 and 2852	–CH (CH_3_)	Stretching asymmetric
(d)	1742	–C=O (ester)	Stretching
(e)	1658	–C=C (*cis*)	Stretching
(f)	1461	–C–H (CH_2_)	Bending (scissoring)
(g)	1378	–C–H (CH_3_)	Bending symmetric
(h) and (i)	1235 and 1161	C–O (ester)	Stretching
(j) and (k)	1118 and 1098	C–O	Stretching
(l)	964	*trans*–CH=CH–	Bending out of plane
(m)	914	*trans*–CH=CH–	Bending out of plane
(n)	871	*trans*–CH=CH–	Bending out of plane
(o)	844	*trans*–CH=CH–	Bending out of plane
(p)	721	*cis*–CH=CH–	Bending out of plane
(q)	1715	–C=O	Stretching

**Table 2:** The composition of fatty acids in NSO, CO, and SO.

Fatty acids	Concentration (%)				
	NSO	CO	CO (Codex)	SO	SO (Codex)
Caproic acid (C6:0)	0.18	ND	ND	0.01	ND
Caprylic acid (C8:0)	0.04	0.00	ND	0.01	ND
Capric acid (C10:0)	0.06	0.00	ND	0.04	ND
Lauric acid (C12:0)	0.07	0.14	ND–0.30	0.02	ND–0.10
Myristic acid (C14:0)	0.02	0.00	ND–0.30	0.06	ND–0.20
Myristoleic acid (C14:1)	0.01	ND	ND	ND	ND
Palmitic acid (C16:0)	19.42	0.08	8.60–16.50	11.29	8–13.50
Palmitoleic acid (C16:1)	0.29	0.001	ND–0.50	0.11	ND–0.20
Oleic acid (C18:1)	45.22	15.32	ND–3.30	0.02	17–30
Linoleic acid (C18:2)	30.18	64.19	34.00–65.60	87.05	48.00–59.00
Linolenic acid (C18:3)	2.07	16.76	ND–2.00	0.64	4.50–11.00
Behenic acid (C22:0)	0.23	ND	ND–0.50	ND	ND–0.70
Eicosatrienoic acid (C20:3)	1.62	0.43	ND	ND	ND
Arachidonic acid (C20:4)	0.59	ND	ND	ND	ND

ND: not detected; the level is below 0.001%.

**Table 3:** PLS performances at some frequency regions for the determination of NSO in binary mixtures with CO.

Frequencies region (cm^−1^)	Spectra	Calibration		Validation	
		*R* ^2^	RMSEC (% v/v)	*R* ^2^	RMSEP (% v/v)
4000–650	Normal	0.9818	3.50	0.9542	3.06
	Derivative 1	0.9904	2.12	0.9975	1.32
	Derivative 2	0.9968	1.61	0.9975	1.15

2977–3028, 1666–1739, 740–1446	Normal	0.9929	2.23	0.9849	2.80
	Derivative 1	0.9933	1.85	0.9970	1.41
	Derivative 2*	*0.9984 *	*1.34 *	*0.9987 *	*0.66 *

1666–1739	Normal	0.9926	2.48	0.9929	1.83
	Derivative 1	0.9966	1.55	0.9950	1.88
	Derivative 2	0.9915	2.08	0.9978	1.21

740–1446	Normal	0.9983	1.11	0.9965	1.34
	Derivative 1	0.9983	1.19	0.9983	0.87
	Derivative 2	0.9921	2.20	0.9952	1.82

*The frequency region and FTIR spectral treatment selected for quantification were marked with italics.

**Table 4:** PLS performances at some frequency regions for the determination of NSO in binary mixtures with SO.

Frequencies region (cm^−1^)	Spectra	Calibration		Validation	
		*R* ^2^	RMSEC (% v/v)	*R* ^2^	RMSEP (% v/v)
4000–650	Normal	0.9918	2.11	0.9927	2.44
	Derivative 1	0.9992	0.73	0.9988	0.59
	Derivative 2	0.9995	0.67	0.9991	0.53

2977–3028, 1666–1739, 740–1446	Normal	0.9995	0.71	0.9991	0.58
	Derivative 1	0.9994	0.75	0.9984	0.66
	Derivative 2*	*0.9995 *	*0.66 *	*0.9994 *	*0.62 *

2985–3024, 752–1755	Normal	0.9993	0.76	0.9992	0.66
	Derivative 1	0.9997	0.47	0.9988	0.63
	Derivative 2	0.9993	0.66	0.9994	0.57

752–1755	Normal	0.9992	0.90	0.9996	0.46
	Derivative 1	0.9993	0.74	0.9997	0.44
	Derivative 2	0.9996	0.59	0.9997	0.64

*The frequency region and FTIR spectral treatment selected for quantification were marked with italics.

**Table 5:** PLS performances at some frequency regions for the determination of NSO in ternary mixtures with CO and SO.

Frequencies region (cm^−1^)	Spectra	Calibration		Validation	
		*R* ^2^	RMSEC (% v/v)	*R* ^2^	RMSEP (% v/v)
4000–650	Normal	0.9975	1.40	0.9986	1.04
	Derivative 1	0.9989	0.99	0.9987	0.76
	Derivative 2	0.9986	0.97	0.9990	0.69

2985–3024; 752–1755	Normal	0.9985	1.07	0.9989	0.873
	Derivative 1	0.9993	0.73	0.9993	0.70
	Derivative 2*	*0.9945 *	*1.77 *	*0.9852 *	*3.02 *

2977–3028; 1666–1739; 740–1446	Normal	0.9987	1.07	0.9984	0.97
	Derivative 1	0.9993	0.80	0.9986	0.78
	Derivative 2	0.9993	0.86	0.9995	0.58

1666–1739	Normal	0.9969	1.56	0.9973	1.27
	Derivative 1	0.9978	1.37	0.9964	1.33
	Derivative 2	0.9971	1.53	0.9970	1.13

740–1446	Normal	0.9704	4.39	0.9777	3.57
	Derivative 1	0.9990	0.88	0.9986	0.89
	Derivative 2	0.9954	1.55	0.9991	0.90

*The frequency region and FTIR spectral treatment selected for quantification were marked with italics.
